# Prevalence and clinical aspects of CMV congenital Infection in a low-income population

**DOI:** 10.1186/s12985-016-0604-5

**Published:** 2016-08-31

**Authors:** Lauro Juliano Marin, Emanuelle Santos de Carvalho Cardoso, Sandra Mara Bispo Sousa, Luciana Debortoli de Carvalho, Marcílio F. Marques Filho, Mônica Regina Raiol, Sandra Rocha Gadelha

**Affiliations:** 1 Health Sciences Department, Universidade Estadual de Santa Cruz, Rodovia Jorge Amado, Km 16, Ilhéus, Bahia Brazil; 2 Department of Biological Sciences, Universidade Estadual de Santa Cruz, Rodovia Jorge Amado km 16, Ilhéus, Bahia Brazil; 3 Department of Biological Sciences, Universidade Estadual do Sudoeste da Bahia, Estrada do Bem Querer, Km 4, Vitoria da Conquista, Bahia Brazil

**Keywords:** Congenital cytomegalovirus disease, Newborn screening, Epidemiology, Late sequelae, Medical supervision

## Abstract

**Background:**

CMV is the most common cause of congenital infection in the whole world (0.2 to 2.2 %). That infection may be symptomatic or asymptomatic at birth and, although asymptomatic cases at birth are more common, some children may develop late sequelae, and require medical intervention. This study aimed to determine the prevalence of CMV congenital infections in children who were born in a public hospital in Ilhéus, Brazil, and to evaluate the clinical progression in infected newborns.

**Methods:**

CMV congenital infection was determined by detecting viral DNA through nested PCR.

**Results:**

The viral DNA was detected in 25 newborns, showing a prevalence of 1.19 % (25/2100) of CMV congenital infection. In regards to the risk factors from mothers, only the variables: age of mothers (*p* = 0.003), number of children (*p* = 0.011), and use of medications (*p* < 0.001) were associated with the congenital infection. Approximately 12 % of children presented symptoms. One death and two auditory alterations were detected during the monitored period. Only 50 % of children diagnosed attended their medical follow.

**Conclusions:**

The prevalence found confirms the findings from other studies which involved other poor populations. Two children presented impaired hearing during the monitored period; that was one of the main sequelae from the infection. It is noteworthy that there was low adherence to medical follow-up which may underestimate data on complications of the infection CMV. Late symptoms can be mistaken for other diseases or even go unnoticed.

## Background

Cytomegalovirus (CMV) infection is common worldwide and its prevalence is inversely proportional to the socioeconomic status of the population and can be higher than 90 % in developing countries including Brazil [1]. In addition, CMV infection has been associated with ethnicity and contact with CMV sources, including people who work in daycare facilities [2, 3].

CMV is the most common cause of congenital infection worldwide and its prevalence varies between 0.2 % and 2.2 % of all infants born [4, 5]. It may be symptomatic, but in 85 – 90 % of the cases, it is asymptomatic at birth. Approximately 90 % of the symptomatic cases involve neurological impairment and unilateral or bilateral sensorineural deafness, with a significant impact on the quality of life, and these cases may progress to death [6, 7]. However, 5 – 15 % of asymptomatic children can present late symptoms and develop progressive, irreversible future sequelae, including impairment of the central nervous system, hearing, and vision, and delayed mental and psychomotor development, among other complications [8, 9].

The diagnosis of the congenital infection should be done before the third week of life because, after this period, it is not possible to assess whether viral transmission occurred through the placenta or through external sources such as the birth canal, saliva, or breast milk [10].

The early diagnosis of CMV congenital infection and clinical follow-up are essential to detect and manage the disease and prevent sequelae [9, 11]. Despite the importance of this infection, many children who are congenitally infected with CMV remain undetected because diagnosis is not performed by the public health system in many countries, including Brazil.

This study aimed to evaluate the prevalence of CMV congenital infections in a public hospital in Ilhéus, Brazil, and its clinical progression during the first years of life.

## Methods

A total of 2100 newborns were included in this study, at any gestational age and in any clinical condition. These newborns were born between February 2010 and December 2012 in Santa Helena Maternity, São José Hospital, located in Ilhéus, southern Bahia, Brazil. Subjects whose parents or guardians refused to sign the consent form were excluded from the study. The study was approved by the Human Research Ethics Committee of the Universidade Estadual de Santa Cruz (UESC) under protocol No. 209/08.

### Sampling

Saliva samples from the newborns were collected with sterile swabs, which were gently placed in their mouth for 1 min to moisten the swabs and transferred to sterile plastic tubes containing 700 μL of transport medium (MEM Earle, Cultilab). After 60 min, the swabs were discarded and the medium was stored at 4 °C until further processing. Urine samples were collected aseptically in hypoallergenic collecting bags. Care was also taken to avoid contamination with meconium. We chose to also use the saliva that has been found to saliva samples seems to be a good specimens for a neonatal screening of congenital CMV due to its ease of collection and for having sensitivity and specificity similar to urine, the gold standard [12].

### Nested PCR

The viral genome was detected via nested PCR in at least two saliva and urine samples obtained before the third week of life. These samples were subjected to PCR without previous DNA extraction and were heated for 6 min at 94 °C. A 2.5 μL-aliquot of saliva or 1.5 μL-aliquot of urine was added to the reaction mixture containing the external primers MIE4-MIE5 [13], a buffer solution (50 mM of KCl and 20 mM of Tris HCl, pH 8.5), 1.5 mM of MgCl_2_, 150 μM of each deoxynucleoside triphosphate; 1U of Taq DNA polymerase (Invitrogen Life Technologies), and 0.3 μM of each primer, adjusted to a final volume of 25 μL. The mixture was subjected to an initial cycle at 94 °C for 2 min, 55 °C for 90 s, and 72 °C for 2 min, followed by 34 cycles at 94 °C for 1 min, 55 °C for 90 s, and 72 °C for 2 min, and an additional extension cycle of 10 min at 72 °C. In the second PCR reaction with the internal primers IE1-IE2, a 0.5 μL-aliquot of each amplified product was used [14]. The reaction mixtures containing the same reagents cited above were subjected to 30 cycles at 94 °C for 1 min, 55 °C for 1 min, and 72 °C for 1 min, followed by an additional extension cycle of 3 min at 72 °C. Positive and negative controls were used. All samples containing viral DNA were retested at least twice using two pairs of primers to confirm the results. The primer pairs used and the size of the amplified fragments are shown in Table 1.

**Table 1:** Sequence of primers used in the PCR for CMV

Primer	Sequence	Product size in base pairs
MIE-4	5’ CAGCACCATCCTCCTCTTCCTCTGG 3’	435
MIE-5	5’ CCAAGCGGCCTCTGATAACCAAGCC 3’	
IE-1	5’ CCACCCGTGGTGCCAGCTCC 3’	159
IE-2	5’ CCCGCTCCTCCTGAGCACCC 3’	

To visualize the PCR products, 10 μL of each amplified product was added to 1.0 μL of GelRed dye and subjected to agarose gel electrophoresis at 2 %. The electrophoresis was conducted at 150 V for 30 min using a Tris/Borate/EDTA buffer solution (0.089 M of Tris, 0.089 M of borate, 0.01 M of EDTA, pH 7.5). Gels were photographed using a digital camera coupled with a UV transilluminator.

### Medical supervision

All children with CMV congenital infections who attended follow-up visits (conducted every three months) were evaluated as follows:

- Clinical examinations, including measurement of weight, height, head and chest circumference, liver size, spleen size, and fundoscopy.
- Complementary examinations: hemogram (including platelet count), liver function test, and measurement of total and fraction bilirubin.
- Hearing assessment.

### Data analysis

To correlate maternal risk factors with the prevalence of CMV infection, the following maternal variables were collected through the application of a questionnaire: age, ethnicity, marital status, number of children, schooling, and use of legal and illegal drugs.

The prevalence of positive cases and symptoms was determined by calculating the prevalence rate.

The epidemiological data and the clinical manifestations in infected and uninfected children were compared. SPSS software version 20.0 was used for statistical analysis. The chi-squared test with Yates’ correction was used whenever applicable. The Kolmogorov-Smirnov test was conducted to assess the normality of quantitative data. P-values lower than 0.05 were considered statistically significant.

## Results

**Fig. 1:**
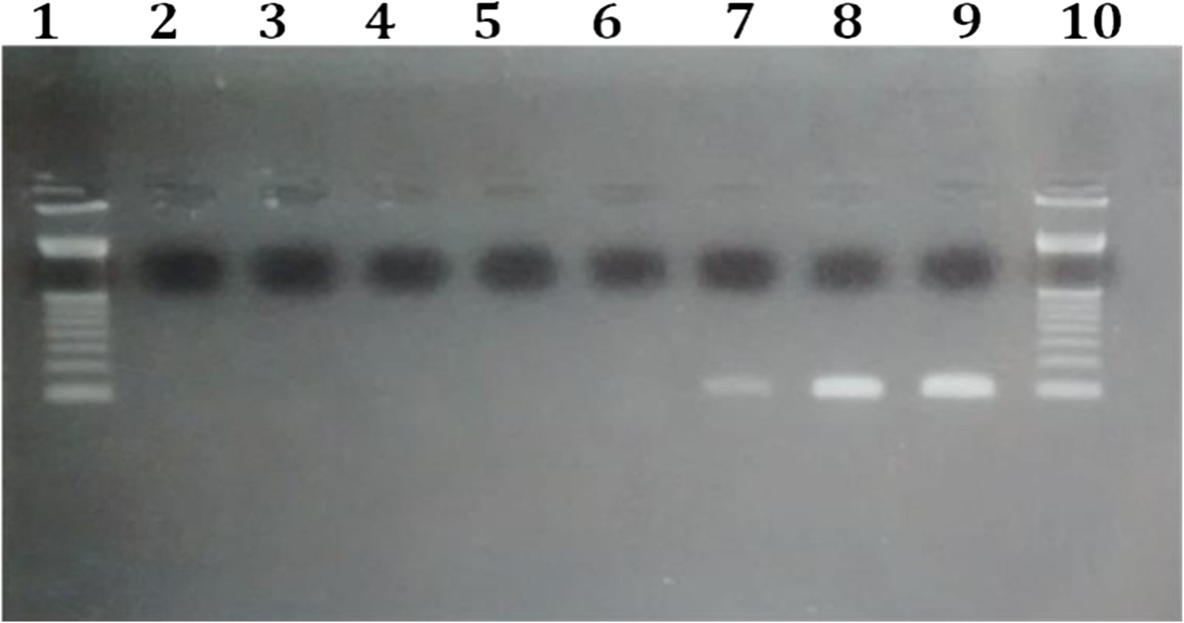
Agarose gel. Agarose gel at 2 % stained with GelRed dye, showing the nested PCR results using urine or saliva samples of 6 newborns during screening for CMV congenital infection. Columns 1 and 10: 100-bp markers; column 2: negative control; columns 3–6: negative samples; column 7: positive sample (159-bp amplicon); columns 8–9: positive controls

Urine and/or saliva samples were collected from 2100 children. Viral DNA was detected in 25 newborns, yielding a prevalence rate of CMV congenital infection of 1.19 % in Ilhéus. The amplified product of 159 bp corresponding to the CMV DNA related to one positive case is presented in Fig. 1.

Only one newborn was symptomatic at birth. However, among the 24 newborns who were clinically diagnosed as asymptomatic, 11 presented one or more general clinical findings that could suggest the presence of cytomegalovirus disease, as shown in Table 2. In order to verify future symptoms in the asymptomatic group, it was conducted a follow-up visits involving complete and periodical clinical examinations of the newborns until the second year of life. During this period, none of the monitored children made use of antiviral drugs.

**Table 2:** Clinical findings suggesting congenital CMV at the time of birth

General clinical aspects	Number of children	%
Low weight	6	25
Prematurity	2	8.3
Petechiae	1	4.17
Bacterial Conjunctivitis	1	4.17
Severe anoxia	1	4.17

The child who presented symptoms at birth died on the seventh day due to cytomegalovirus disease. This female infant was born by normal delivery in a 36-week pregnancy, weighed 2,095 g, had a head circumference of 24 cm, chest circumference of 27.5, and body length of 44 cm. The clinical changes during disease progression included discomfort while breathing, globular and flaccid abdomen, generalized edema, jaundice, tachypnea, petechiae, sloughing, and weak reflexes. The laboratory alterations included C-reactive protein levels of 48 mg/dL (reference value of 0.1 mg/dL), marked cell atypia, serum glutamic oxaloacetic transaminase (GOT) levels of 78 URF/mL (reference value of 4–36 URF/mL), and thrombocytopenia.

Only 12 children (50 %) attended the periodic follow-up visits. Of these, only 2 children (16.7 %) presented late CMV symptoms (auditory alterations) and were referred to brainstem-evoked response audiometry (BERA). However, both children needed to relocate and were no longer monitored.

In relation to epidemiological characteristics of the studied population according to CMV infection positivity, the age group of 12–15 years presented the highest number of children with CMV congenital infection (*p* = 0.033). In addition, out of the 25 mothers of infected newborns, 17 were primiparae (*p* = 0.011) (Table 3). There was no statistically significant correlation between the data on delivery and the characteristics at birth of the infected newborns (Table 4).

**Table 3:** Clinic and epidemiological characteristics of mothers according to CMV infection

	Mothers of CMV infected children (*n* = 25)	Mothers of CMV no infected children (*n* = 2075)	*P*-value
12 to 15 years old	4 (16 %)	124 (6 %)	0.033
>5 sexual partners	20 (80 %)	1773 (85.4 %)	0.418
Sexual onset before 19 years old	24 (96 %)	1776 (85.6 %)	0.546
First child	17 (68 %)	456 (22 %)	0.011
Close contact with children < 2 years old	10 (40 %)	1108 (53.4 %)	0.506
Other infectious disease	0 (0.0 %)	230 (11.1 %)	
Genitourinary disease	0 (0.0 %)	45 (2.2 %)	

Values of *p* < 0.05 are considered significant

**Table 4:** Data from deliveries and clinical characteristics of children according to CMV infection

	CMV-positive (25)	CMV-negative (2075)	*P*-value
Caesarean section	9 (36 %)	1056 (50.9 %)	0.335
Normal delivery	14 (56 %)	997 (48 %)	
Normal weight at birth	20 (80 %)	1799 (86.7 %)	0.268
Low body weight	4 (16 %)	162 (7.8 %)	
Premature	1 (4 %)	97 (4.7 %)	0.642
Term birth	22 (88 %)	849 (41 %)	
Post-term birth	0 (0.0 %)	1 (0.05 %)	
Normal head circumference	19 (76 %)	1252 (60.3 %)	0.423
Decreased head circumference with deficiency	2 (8 %)	57 (2.7 %)	
Increased head circumference	2 (8 %)	194 (9.3 %)	

Values of *p* < 0.05 are considered significant

## Discussion

The prevalence of 1.19 % found in Ilhéus was similar to the findings of other studies conducted in low-income regions. In fact, the prevalence varied between 0.2 % and 2.2 % depending on the region and on the socioeconomic status of the population [5, 7, 15–19].

With regard to the symptoms, 87.5 % of the newborns were asymptomatic and 12.5 % showed symptoms. One of them showed symptoms at birth and two of them showed late symptoms detected during follow-up. The child who presented symptoms at birth died on the seventh day from cytomegalovirus congenital disease, which was confirmed by molecular diagnosis and by laboratory and clinical tests. This newborn was pre-term (less than 37 weeks), had low-birth weight (below the 10th percentile of the intrauterine growth curve), decreased head circumference with a trace of microcephaly (below the 10th percentile of the intrauterine growth curve), petechiae, globular abdomen, discomfort while breathing, generalized edema, jaundice, tachypnea, sloughing, and weak reflexes. Laboratory examinations indicated altered C-reactive protein levels and increased GOT.

The two children with late symptoms showed auditory alterations characterized by the lack of auditory response detected at 2 years of age, and were referred to BERA. Previous studies have reported varying rates of hearing loss associated with CMV congenital infection, time of clinical follow-up, and primary maternal infection during pregnancy [7, 20].

Only 12 mothers or guardians attended the previously scheduled post-natal follow-up visits. Some mothers discontinued the supervision for different reasons including changed telephone numbers or changed addresses. The high number of absences in follow-up visits may mask the infection symptomatology and even prevent those children from receiving proper care. Furthermore, late onset symptoms related to CMV infection may be mistaken for other diseases or even go unnoticed. The difficulty to reach patients confirms the importance of creating a health education program to educate the population about these infections and the strategies to prevent and treat them.

The maternal age and number of children were associated with CMV congenital infection. In addition, infection was more frequent in young mothers (12 – 15 years old). The early sexual maturity and lack of information concerning CMV infection may increase the risk of primary infections, which lead to a higher likelihood of vertical virus transmission as demonstrated in the literature [21, 22]. Moreover, the present study showed that most young mothers were primiparous and that primiparous women had higher likelihood of transmitting the infection vertically, thereby contributing to the increased infection rate among the newborns.

## Conclusions

The prevalence of CMV infection in Ilhéus, Brazil, was similar to the findings of other studies conducted in low-income regions and most infected children were asymptomatic. However, late symptoms related to CMV can occur, leading to progressive and irreversible sequels. Besides, the high number of absences in follow-up visits observed in this study may mask the infection symptomatology and even prevent those children from receiving proper care. It is noteworthy that the implementation of a national educational policy and the monitoring of infected children are important strategies aimed to decrease the risk of virus transmission and detect late symptoms and sequelae.

## Abbreviations

BERA: Brainstem-evoked response audiometry
CMV: Cytomegalovirus
GOT: Glutamic oxaloacetic transaminase
UESC: Universidade Estadual de Santa Cruz
